# Mesenchymal stem cells with Sirt1 overexpression suppress breast tumor growth via chemokine-dependent natural killer cells recruitment

**DOI:** 10.1038/srep35998

**Published:** 2016-10-26

**Authors:** Yang Yu, Yan Liu, Chen Zong, Qingbo Yu, Xue Yang, Lei Liang, Fei Ye, Li Nong, Yuxian Jia, Yongkui Lu, Zhipeng Han

**Affiliations:** 1 Department of Urology, Affiliated Tumor Hospital of Guangxi Medical University, Nanning, People’s Republic of China; 2 The Fifth Department of Chemotherapy, Affiliated Tumor Hospital of Guangxi Medical University, Nanning, People’s Republic of China; 3 Tumor Immunology and Gene Therapy Center, Eastern Hepatobiliary Surgery Hospital, the Second Military Medical University, Shanghai, People’s Republic of China; 4 Department of Thyroid Breast Surgery, Affiliated Hospital of Weifang Medical University, Weifang, People’s Republic of China

## Abstract

Mesenchymal stem cells (MSCs) are generally used in regenerative medicine, tissue engineering and therapy for immune disorder diseases. However, due to the immunosuppressive function of MSCs, the application of MSCs in breast cancer therapy remains limited. Sirt1 is the closest mammalian homologue of the yeast enzyme Sir2 which has an established capacity to influence yeast replicative lifespan. In this study, we demonstrated the effect of MSCs with Sirt1 overexpression (MSCs-Sirt1) in mice bearing 4T1 breast cancer and investigated the underlying mechanism. Firstly, we found that MSCs could accelerate breast tumor growth with promoted proliferation and inhibited apoptosis, whereas MSCs-Sirt1 significantly suppressed tumor growth with proliferation inhibition and apoptosis promotion. Moreover, we detected that NK cells were the prominent antitumor effectors for the MSCs-Sirt1-induced antitumor activity. Besides that, CXCL10 and IFN-γ showed the high level expression in MSCs-Sirt1 treatment group. The impulsive effect of MSCs-Sirt1 on 4T1 cells *in vivo* could be reversed by inhibition of CXCL10 and IFN-γ. Overall, our results suggest that MSCs-Sirt1 can effectively inhibit breast tumor growth via the recruitment of NK cells in tumor inflammatory microenvironment.

Breast cancer is a leading cause of mortality among women with cancer in the United States^1^, and shows an increasing incidence in the developing world. It has been reported that breast cancer is the most commonly diagnosed cancers among women in China, which is expected to account for 15% of all new cancers in women^2^. As a heterogeneous disease with distinct molecular subtypes, breast cancer has very different prognoses. Especially hormone-independent and triple-negative carcinomas are problematic due to the limited options for providing adjuvant therapy^3^. It is essential to find a new effective method for breast cancer therapy.

Mesenchymal stem cells (MSCs) are a heterogeneous subset of stromal stem cells, which can be isolated from bone marrow^4^. MSCs may differentiate into various specialized cell types under certain physiological or experimental conditions, which is a potential source of stem cells for cellular and genetic therapy^5^. And based on its low immunogenicity, MSCs are believed to be a promising stem cell population for clinical applications, especially in treating immune-based disorders^6^,^7^,^8^. In recent years, MSCs have been attempted to use for prevention or treatment with autoimmune diseases, such as experimental autoimmune encephalomyelitis and collagen-induced arthritis^9^,^10^. Besides that, the immunomodulatory effect of MSCs is plastic, depending on the inflammatory status of tissue microenvironment^11^,^12^. Currently, there are many studies showing that MSCs can migrate to injured tissues and induce peripheral tolerance, where they can inhibit the release of pro-inflammatory cytokines and promote the survival of damaged cells^13^,^14^,^15^. MSCs from bone marrow have also been shown to be an important component of the tumor microenvironment, assisting tumor escape from immunosurveillance^16^, which contributes to the growth of cancer cells. Nowadays, it has been demonstrate that MSCs could migrate into the breast cancer tissue and play a significant role in breast cancer development^17^,^18^.

Sirtuins is a molecular family with seven members (Sirt1–7), of which Sirt1 is the closest mammalian homologue of the yeast enzyme Sir2, a protein with an established capacity to influence yeast replicative lifespan^19^. Consequently, the tremendous interest in Sirt1 occurred rapidly due to its possible role in eukaryote. It has been proved that Sirt1 plays an important role in regulating several biological functions, such as aging, metabolism, DNA damage and tumor development in mammalian^20^. Sirt1 was also shown to be expressed in MSCs^21^ and the essential roles of Sirt1 in the proliferation and differentiation of MSCs have gain more interest in recent years^22^. It has been reported that overexpression of SIRT1 in aged MSCs could reverse the senescence phenotype and stimulated cell proliferation. However, the exactly effect of mammalian Sirt1 overexpressed MSCs on cancer as a metabolic and age-related disease remain unclear.

In this study, we constructed Sirt1 overexpressed MSCs (MSC-Sirt1) through infecting MSCs with an adenovirus containing the Sirt1 gene and used the 4T1 breast cancer cell line to observe the potential effect of MSC-Sirt1 on regulating breast cancer cells growth *in vivo*. We further investigated the mechanism underlying the regulatory effects of MSCs-Sirt1 on tumor growth. This report shows that MSCs-Sirt1 can exert a profound inhibitory effect on tumor growth and this would be a new potential manner for breast cancer therapy.

## Results

### MSCs with Sirt1 overexpression can effectively suppress breast tumor growth

**Figure 1:**
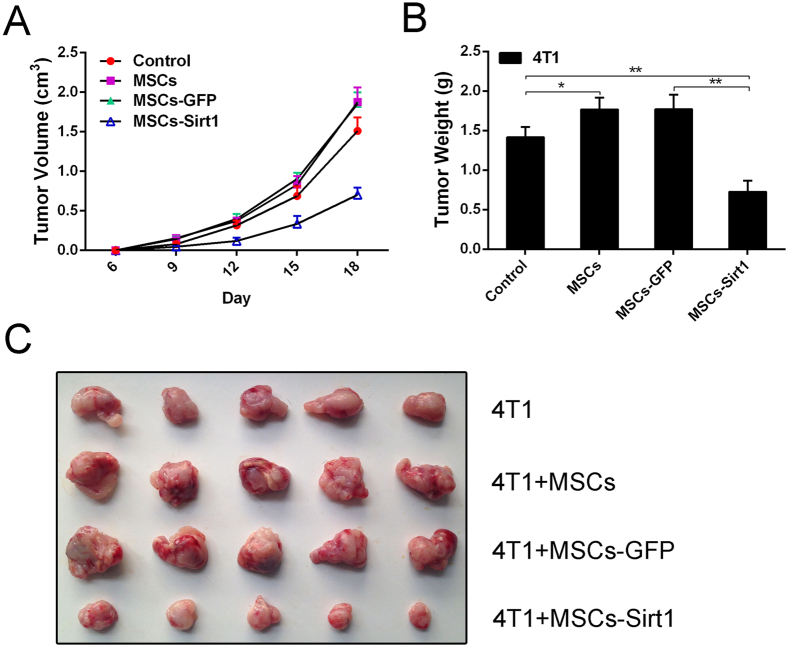
The effect of MSCs with Sirt1 overexpression on breast cancer tumor growth in mice. (**A**) The tumor growth were detected per 3 days after subcutaneous administration with 4T1 cells. The width and length of 4T1 tumors were measured, then the volume of tumor was calculated using the formula: volume = width^2^  × length × 0.5236. (**B**) Tumor weights were measured after they were harvested from the mice. (**C**) Tumors were presented by representive photographs. Each group consists of 5 mice. *, p < 0.05; **, p < 0.01. MSCs-GFP, MSCs with GFP overexpression; MSCs-Sirt1, MSCs with Sirt1 overexpression.

Firstly, bone marrow-derived MSCs were prepared and detected as previous study^23^. To demonstrate the effect of MSCs with Sirt1 overexpression, we constructed Sirt1 overexpressed MSCs through infecting MSCs with an adenovirus containing the Sirt1 gene (Supplementary Fig. 1). Then, we applied subcutaneously implanted tumor model in BALB/c mice to determine the effect of MSCs-Sirt1 on 4T1 breast cancer cells growth *in vivo*. Mice were treated as described in materials and methods. All mice survived until they were sacrificed at 18^th^ day after subcutaneous injection. As shown in Fig. 1A, the tumor volume increased at an accelerated rate. The development of 4T1 cells coinjected with MSCs was significantly faster than those injected alone, while 4T1 cells coinjected with MSCs-Sirt1 grew the most slowly. Besides that, tumors in weight were more heavier in the group coinjected with MSCs and 4T1 cells than those in the group injected with 4T1 cells alone. However, when 4T1 cells were coinjected with MSCs-Sirt1, tumor showed a significant reduction in weight (Fig. 1B,C). These results indicate that MSCs-Sirt1 can inhibit the growth of breast cancer in mice.

### MSCs with Sirt1 overexpression could induce tumor cells apoptosis promotion and proliferation inhibition *in vivo*

**Figure 2:**
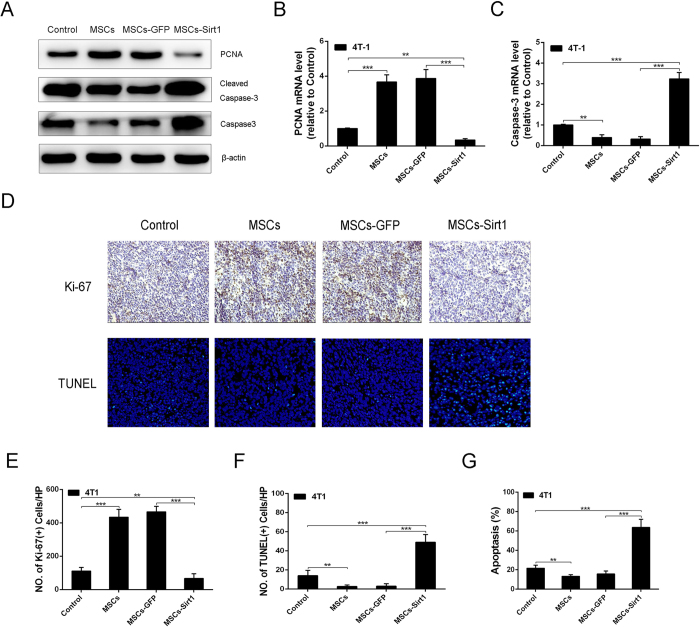
The effect of Sirt1 overexpressed MSCs on 4T1 cells prolifeation and apoptosis *in vivo.* (**A**) Western blotting was employed to examine the PCNA, Caspase-3 and Cleaved Caspase-3 protein expression levels of tumors. Real-time PCR was employed to examine the PCNA (**B**) and Caspase-3 (**C**) expression levels of tumors. Results were reported as ratio to control group. (**D**) 4T1 tumor tissues were analyzed for immunohistochemical staining of Ki-67. Apoptosis of 4T1 tumor tissues were determined by TUNEL assay. Typical photographs were presented (original magnification: × 200). **(E**) The number of Ki-67-positive cells per HP (mean ± SD) was quantified from each group. (**F**) The quantification of TUNEL-positive tumor cells was shown as mean ± SD. (**G**) The apoptosis was analyzed by flow cytometry using Annexin V-FITC/PI and data were shown as mean ± SD. HP, high power field (400 × ). **, P < 0.01; ***, P < 0.001.

To evaluate the tumor cells proliferation and apoptosis induced by MSCs-Sirt1, the protein expression of PCNA (a cell proliferation indicator) and Caspase-3 (a cell apoptosis indicator), as well as mRNA expression were measured by western blotting and real-time PCR respectively. As shown in Fig. 2A–C, MSCs treatment groups induced a higher expression level of PCNA and a lower expression level of Caspase-3 (or Cleaved Caspase-3) than those of control groups. In contrast, MSCs-Sirt1 treatments resulted in a decrease in the expression level of PCNA and an increase of Caspase-3 (or Cleaved Caspase-3) compared with the controls (Fig. 2A–C).

We also analyzed tumor xenograft tissues sections with Ki67 and TUNEL, markers for proliferative and apoptotic response respectively. As shown in Fig. 2D–F, compared with controls, MSCs treatment groups showed a marked increase in number of Ki67-positive cells and an obvious decrease in number of TUNEL-positive cells. Conversely, there was an apparent rise of the percentage of TUNEL-positive tumor cells and a significant decline of Ki67-positive tumor cells in MSCs-Sirt1 treatment groups (Fig. 2D–F). Additionally, FACS analysis showed the corresponding data for apoptosis (Fig. 2G). Together, these results indicate that MSCs-Sirt1 can suppress breast tumor growth through proliferation inhibition and apoptosis induction.

### Sirt1 overexpressed MSCs treatment elicits significantly high level of IFN-γ *in vivo*

**Figure 3:**
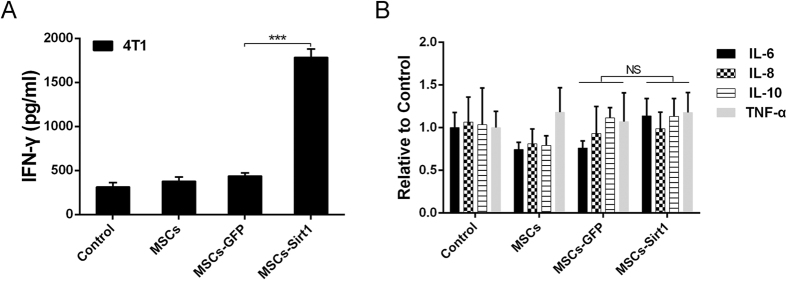
Serum inflammatory cytokines levels of mice suffered with subcutaneous tumors. (**A,B**) Serum inflammatory cytokines levels were determined by Bio-Plex Pro^TM^ mouse cytokine assay kit (Bio-Rad Laboratories, USA) at 18^th^ day since cells subcutaneous injection. Each group consists of 5 mice. ***, P < 0.001; NS, P > 0.5.

Several studies have reported that MSCs can enhance tumor development through immunosuppressive activity with inhibiting the release of pro-inflammatory cytokines^13^,^14^,^15^. Then we detected related inflammatory cytokines including IL-6, IL-8, IL-10, IFN-γ, TNF-α etc. Interestingly, MSCs-Sirt1 treatment groups had higher IFN-γ level of serum in mice than other treatment groups (Fig. 3A). Meanwhile, there was no obvious difference in other inflammatory cytokines (Fig. 3B). These data suggest that MSCs-Sirt1 can greatly induce IFN-γ production *in vivo*.

### Sirt1 overexpressed MSCs recruit natural killer cells to tumor tissues for tumor suppression

**Figure 4:**
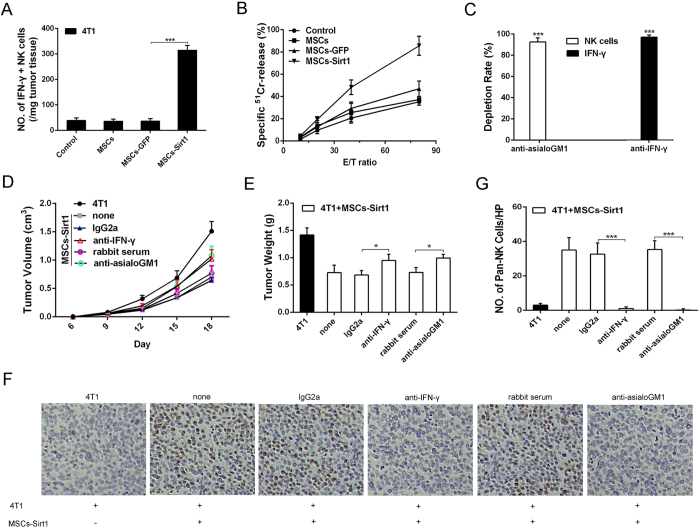
The effect of NK cells on tumor repression in tumor-bearing mice. (**A**) Tumor infiltrating cells were isolated from the tumors of mice and were stained with antibody against NK cells, followed by intracelluar IFN-γ staining. (**B**) Splenocytes (effector, E) were isolated from the mice after sacrifice and assayed against YAC-1 cells (target cell, T). Cell lysis was determined in triplicate and ^51^Cr isotope release assay at different effector-to-target cell (E/T) ratios were performed to determine the cytotoxic potential of effector populations. (**C**) Mice were depleted of IFN-γ and NK cells by anti-IFN-γ mAb and rabbit anti-asialoGM1 antiserum by intraperitoneal injection, respectively. The depletion rates were also texted through ELISA kit and flow cytometry. Each group consists of 5 mice. (**D**) 4T1 tumor growth under IFN-γ or NK cell subset depletion was examined again. Mice treated with an irrelevant mouse monoclonal IgG2a or normal rabbit serum at the same dose were included as control. The width and length of 4T1 tumors were measured per 3 days, then the volume of tumor was calculated using the formula: volume = width^2^  × length × 0.5236. (**E**) Tumor weights were measured after they were harvested from the mice. (**F**) Tumor tissues were analyzed for immunohistochemical staining of CD49b for pan-NK cells (original magnification: × 200). (**G**) The number of pan-NK cells per HP (mean ± SD) was quantified. Each group consists of 5 mice. HP, high power field (400 × ). *, P < 0.05; ***, P   < 0.001.

Natural killer (NK) cells are important immune cells, which can produce many inflammatory cytokines. Moreover, NK cells are the major IFN-γ-secreting cells *in vivo*^24^. In order to confirm whether NK cells induced local inflammatory response and performed the effect of MSCs-Sirt1-induced tumor suppression in tumor-bearing mice, tumor-infiltrating NK cells were isolated and analyzed by flow cytometry. As shown in Fig. 4A, dramatically higher numbers of IFN-γ-secreting NK cells were detected in tumor xenograft tissues of MSCs-Sirt1 group than those in tumor xenograft tissues of control group, as well as MSCs-GFP group. However, tumor xenograft tissues of MSCs group showed lower numbers of IFN-γ-secreting NK cells than those of control group. Besides that, we isolated splenocytes from the mice to detect the cytolytic NK activity. The data showed that there was a significant enhancement of cytolytic NK activity in MSCs-Sirt1 treatment group compared with those in other treatment group (Fig. 4B). These results were in good agreement with the finding presented in Fig. 4A.

Next, to further clarify the role of IFN-γ and NK cells in MSCs-Sirt1-induced immunopotentiation to inhibit breast tumor growth *in vivo*, anti-asialoGM1 antiserum was utilized to inhibit the production of NK cells, and anti-IFN-γ antibody was also applied to realize IFN-γ depletion in tumor-bearing mice. As depicted in Fig. 4C, 97.1% depletion for IFN-γ and 92.6% depletion for NK cells were measured by ELISA and flow cytometry respectively. Obviously, as shown in Fig. 4D,E, neutralization of IFN-γ greatly impaired the antitumor effect of MSCs-Sirt1 in tumor-bearing mice and the tumor growth was greatly restored. Similarly, depletion of NK cells significantly weakened the antitumor effect, establishing that NK cells were critical to the antitumor effects of MSCs-Sirt1 in tumor-bearing mice. Then, we analyzed tumor xenograft tissues sections with CD49b, marker for NK cells. There was a significant decline of pan-NK cells when neutralization of IFN-γ or NK cells was performed in MSCs-Sirt1 group (Fig. 4F,G). Together, these results suggest that MSCs-Sirt1 can perform antitumor effect via NK cells recruitment.

### Sirt1 overexpressed MSCs promote NK cells recruitment by increasing CXCL10 expression *in vivo* and *in vitro*

**Figure 5:**
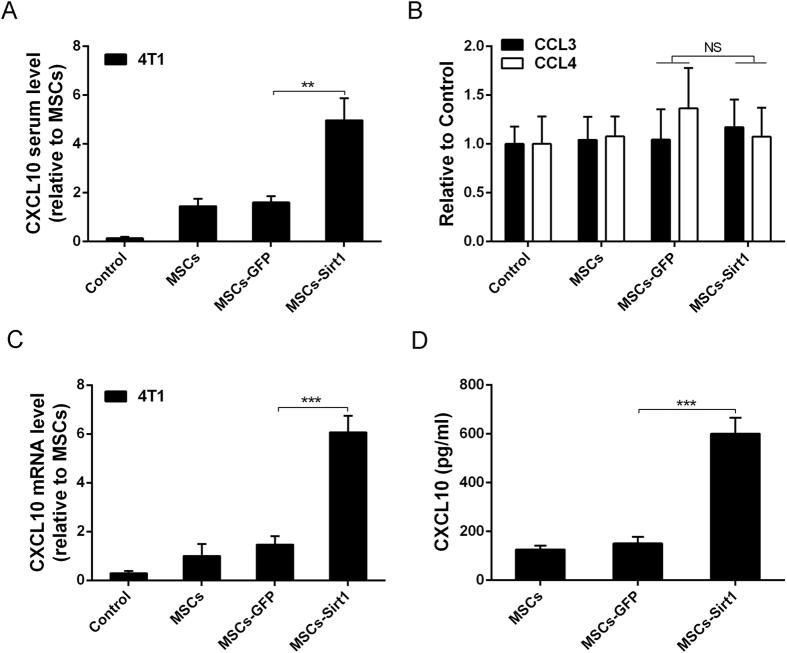
The evaluation of chemotactic factors production. ELISA kit was utilized to determine the serum chemotactic factors level of tumor-bearing mice, including CXCL10 (**A**), CCL3 and CCL4 (**B**). Results were reported as ratio to MSCs group. Serum samples were taken at the 18^th^ day since cells subcutaneous injection. Each group consists of 5 mice. (**C**) Real-time PCR was employed to examine the CXCL10 expression level of tumors and the data were reported as ratio to MSCs group. Each group consists of 5 mice. (**D**) ELISA assay was also used to examine the CXCL10 expression in the conditioned medium of MSCs, MSCs-GFP and MSCs-Sirt1. The data presented are from three replicates as mean ± SD. **, P < 0.01; ***, P < 0.001; NS, P > 0.5.

To detect the potential mechanism of MSCs-Sirt1 recruiting NK cells for tumor inhibition, the level of related chemotactic factors which could recruit NK cells including CCL3, CCL4 and CXCL10 were examined in tumor-bearing mice serum through ELISA. As shown in Fig. 5A, the serum CXCL10 level was effectively upregulated in MSCs-Sirt1 group compared with that in MSCs-GFP group. Meanwhile, there was no obvious difference in the other two chemotactic factors (Fig. 5B). Next, we found that MSCs-Sirt1 treatments showed a marked increase on mRNA expression level of CXCL10 *in vitro* compared with MSCs-GFP treatments (Fig. 5C). Besides that, MSCs-Sirt1 showed a higher expression of CXCL10 *in vitro* than MSCs-GFP (Fig. 5D). All of these results imply that CXCL10 may be the key chemotactic factor that recruits NK cells for antitumor effect.

### Sirt1 overexpressed MSCs perform breast tumor inhibition through CXCL10-recruited NK cells *in vivo*

**Figure 6:**
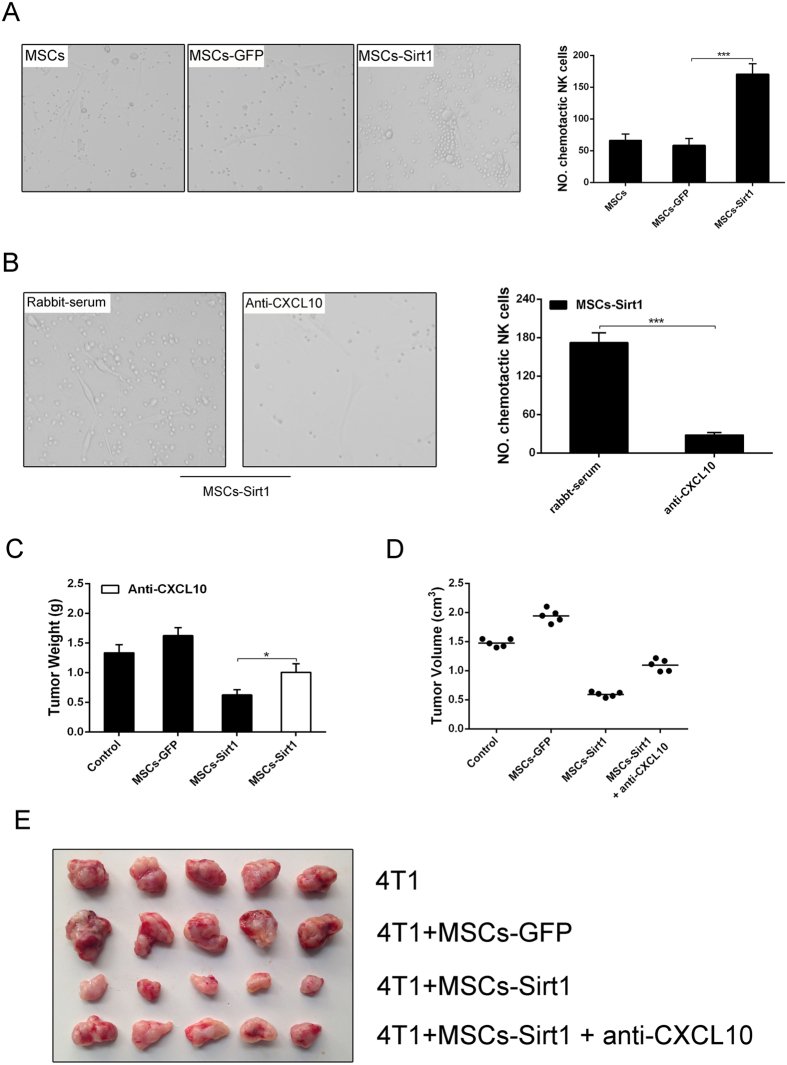
CXCL10 recruits NK cells caused subcutaneous tumor suppression. (**A**) Conditioned medium of MSCs, MSCs-GFP, and MSCs-Sirt1 were applied to detect their chemotactic effect to NK cells via transwell assay. NK cells were counted under a microscope. Typical photographs were presented (original magnification:  × 200). Mean level was obtained for plotting. The data presented are from three replicates as mean ± SD. (**B**) The role of chemotactic factor CXCL10 in NK cells migration were demonstrated through rabbit anti-murine CXCL10 (Abnova) *in vitro*. MSCs-Sirt1 treated with an irrelevant normal rabbit serum at the same dose were included as control conditioned medium. NK cell count was obtained as described above. 4T1 tumor weight (**C**) and volume (**D**) under CXCL10 depletion were examined in mice treated with rabbit anti-murine CXCL10 (2.5 ug/5ul, Abnova) intraperitoneally. Each group consists of 5 mice. (**E**) Tumors were presented by representive photographs. *, P < 0.05; ***, P < 0.001.

To confirm the CXCL10 chemotactic effect on NK cells, we further performed transwell co-culture of the NK cells and MSCs-Sirt1 *in vitro*. Results showed that MSCs-Sirt1 had a strongly chemotactic effect on NK cells compared with MSCs-GFP (Fig. 6A). Whereas MSCs-Sirt1 treated with rabbit anti-murine CXCL10 could attenuate the chemotactic effect on NK cells (Fig. 6B). These results demonstrate that MSCs-Sirt1 can effectively recruit NK cells through CXCL10 secretion.

In order to further elucidate the role of CXCL10 in tumor inhibition, rabbit anti-murine CXCL10 was also applied to the tumor-bearing model. BALB/c mice were injected intraperitoneally with rabbit anti-murine CXCL10 for CXCL10 depletion or rabbit-serum as control before the treatment of MSCs-Sirt1 and 4T1 cells subcutaneous coinjection. Dramatically, the tumor showed an increased weight and volume when rabbit anti-murine CXCL10 was additionally injected in tumor-bearing mice with MSCs-Sirt1 and 4T1 cells coinjection, compared with those under rabbit-serum injection (Fig. 6C–E). The data indicate that the effect of MSCs-Sirt1 in tumor suppression can be greatly blocked by CXCL10 inhibition. These results demonstrate that CXCL10 can recruit NK cells contributing to the MSCs-Sirt1 induced suppression of breast tumor growth in mice.

## Discussion

Mesenchymal stem cells, as a heterogeneous subset of stromal stem cells, can differentiate into various specialized cell types^5^ and they have been recognized to contribute to the regeneration of a wide variety of organs and healing of some diseases^25^,^26^,^27^. In recent years, several studies have reported that MSCs play an important role in immunosuppressive effects^6^,^7^,^8^. Unfortunately, tumor can create a tumor microenvironment promoting tumor development through escaping immune surveillance^28^,^29^, thus the immunosuppressive effect may promote tumor growth in certain circumstances. Djouad *et al*. revealed that MSCs exhibited side effects related to systemic immunosuppression which induced tumor growth *in vivo*^13^. Several previous studies have also clarified that MSCs can be recruited to breast cancer site and accelerate the cancer growth and invasion^17^,^18^,^30^. So there are still various problems limiting the application of MSCs in clinical therapy. Therefore, we designed the current study to investigate the effect changes of MSCs on breast tumor growth after they were infected with the Sirt1 overexpression. Interestingly, the major finding of this study was that Sitr1 overexpressed MSCs showed a strong suppressive effect on 4T1 breast cancer cells growth *in vivo*, in contrast to the promoting effect of MSCs in tumor-bearing mice model. Furthermore, we demonstrated that MSCs-Sirt1 could induce apoptosis and inhibit proliferation to suppress 4T1 tumor growth *in vivo*. We then proved for the first time that this antitumor effect was associated with the enhancement of inflammatory responses.

As we know, reinforcing the function of the immune system could effectively control cancer progression. Several reports have demonstrated that intratumoral infiltration of NK cells correlates with a good prognosis in a variety of cancers^31^,^32^,^33^. NK cells constitute an important component of the innate immune system, performing surveillance against intracellular bacteria, certain viruses, and transformed cells^34^. NK cells can be rapidly activated to attack certain abnormal cells spontaneously through producing large inflammatory cytokines (eg: TNF-α, IFN-γ etc.) and chemotactic factors (eg: CCL3, CCL4, CXCL10 etc.) *in vivo*^24^, especially tumor or virus infected cells, so lack of NK cells increased susceptibility to viral infections and possibility to tumorigenesis^35^. In recent years, adoptive infusion of NK cells has gained an increased attention as immunotherapy against cancer^36^. In the current study, we detected high level of IFN-γ-secreting NK cells in tumor tissues excised from mice under MSCs-Sirt1 and 4T1 cells coinjection, compared with those under MSCs-GFP and 4T1 cells coinjection, as well as 4T1 cells injection alone. Then we made a depletion on NK cells in mice and the result showed that the antitumor effect of MSCs-Sirt1 was obviously weakened *in vivo*, which indicated that NK cells played an important role in tumor inhibition induced by MSCs-Sirt1 in tumor-bearing mice.

Consistently, we detected significantly higher IFN-γ level of mice serum in MSCs-Sirt1 treatment group than those in other different treatment groups and the antitumor effect of MSCs-Sirt1 in tumor-bearing mice was greatly impaired when neutralization of IFN-γ was performed, which suggested that IFN-γ showed an important effect on MSCs-Sirt1-induced tumor inhibition in tumor-bearing mice. However, whether the antitumor effect of MSCs-Sirt1 performed through NK cells secreting IFN-γ is unidentified and it is pending further investigation. CXCL10 is an inflammatory chemokine produced by different cell types such as endothelial and epithelial cells, as well as keratinocytes, in response to IFN-γ^37^. It is related to NK cells migration and involved in inflammatory processes^37^. On one hand, our data demonstrated an obvious increase of CXCL10 expression in serum level and mRNA level *in vivo* when the serum and tumors of tumor-bearing mice were harvested at the end of the experiment. In addition, we also proved that MSCs-Sirt1 showed a significant increase in CXCL10 production, which performed a powerful chemotaxis effect on NK cells *in vitro*. On the other hand, as we know CXCL10 is a chemotactic factors which can also be produced by NK cells^24^,^38^, which plays important biological function in promoting immune responses and antitumor effects^39^. In our study we evaluated the effect of CXCL10 via neutralization, which demonstrated that CXCL10 played a significant role in MSC-Sirt1-induced tumor suppression *in vivo*. Overall, all of our findings support that the increased production of inflammatory cytokines and chemokines maintain MSCs-Sirt1-induced inflammatory responses with consequently tumoricidal activation. However, the source of CXCL10 in tumor-bearing mice need a further study.

In conclusion, in view of the limitation of MSCs in cancer therapy, it is necessary to explore a new manner to make MSCs play negative effect on tumor growth. Significantly, we infected MSCs with an adenovirus containing the Sirt1 gene and demonstrated the antitumor function of MSCs with Sirt1 expression through enhancing local inflammatory responses induced by NK cells in this study. Our preliminary results suggest that MSCs-Sirt1 may represent a promising strategy for tumor treatment in the clinic.

## Materials and Methods

### Cell culture

MSCs were generated from bone marrow flushed out of tibia and femur of 4–6 weeks old BALB/c mice. Cells were cultured in DMEM medium supplemented with 10% fetal bovine serum (FBS), 2 mM glutamine, 100 U/ml penicillin, and 100 mg/ml streptomycin (all from Invitrogen, Carlsbad, CA) as previous report^23^. Murine 4T1 cells were cultured in RPMI-1640 medium with 10% FBS, supplemented with 2 mM L-glutamine, 100 U/ml penicillin, and 100 mg/ml streptomycin. Mouse spleens were disaggregated into 10 ml RPMI-1640 medium to isolate splenocytes. Murine NK cells were prepared by NK Cell Activation/Expansion Kit (Miltenyi Biotec) and cultured in RPMI-1640 medium supplemented with 10% FBS. All cells were cultured at 37 °C in a 5% CO_2_ humidified atmosphere.

### Adenoviral vector construction

The adenoviral vector containing a mouse Sitr1 cDNA (Ad-Sirt1) or a GFP gene (Ad-GFP) as control was constructed. According to the manufacturer’s instruction, the recombined adenoviral expression vectors were constructed by the Gateway Cloning System (Invitrogen, Carlsbad, CA, USA). Cells were observed under a fluorescence microscope. By verification, we confirmed that adenoviral delivery of Sirt1 (Ad-Sirt1) are effect on 48^th^ hour, and then the next experiments were performed.

### Animals and treatments

Female BALB/c mice, 6–8 weeks old, weighting 21–23 g, were obtained from the Shanghai Experimental Animal Center of the Chinese Academy of Sciences, Shanghai, China. Mice in this study were housed in pathogen-free conditions. All animal experiments were approved by the Animal Care and Experimentation Committee of Affiliated Tumor Hospital of Guangxi Medical University, and in accordance with the guidelines of Affiliated Tumor Hospital of Guangxi Medical University. The mice were divided randomly into four groups (n = 5/group) and builded tumor-bearing model closely mimicking the tumor growth of breast cancer. MSCs after infection 48 hours were utilized for tumor formation. Subcutaneous administration of these cells was performed in the armpit area of mice, separately defining as Control group (1 × 10^6^ 4T1 cells in 200 μl PBS), MSCs group (1 × 10^6^ 4T1 cells and 2 × 10^5^ MSCs in 200 μl PBS), MSCs-GFP group (1 × 10^6^ 4T1 cells and 2 × 10^5^ MSCs-GFP in 200 μl PBS), and MSCs-Sirt1 group (1 × 10^6^ 4T1 cells and 2 × 10^5^ MSCs-Sirt1 in 200 μl PBS). Mice were observed every 3 days to detect tumor formation and tumor growth was evaluated by measuring the length and width of tumor mass using calipers. All tumor-bearing mice were sacrificed on the 18^th^ day. At the end of the experiment, blood samples were collected and spleens were excised for further experiment. Tumor tissues were then collected and photographed. the length, width and weight of tumors were measured. Tumor tissues were also dissected quickly, and portions of tissue were fixed in neutral buffered formalin, while others were frozen in liquid nitrogen immediately and stored at −80 °C. Serum samples were obtained by centrifugation (1750g, 15 min, 4 °C) and stored at −80 °C for further analysis.

### Flow cytometric analysis

Tumor-infiltrating lymphocytes from breast cancer tumor tissues were evaluated by flow cytometric analysis. At first, tumor tissues were briefly cut into small pieces using a razor blade and the tissue fragments were incubated for 15 minutes at 37 °C using HBSS solution containing collagenase type I (0.05 mg/ml), collagenase type IV (0.05 mg/ml), hyaluronidase (0.025 mg/ml), DNase I (0.01 mg/ml) and soybean trypsin inhibitor (1mg/ml) (Sigma). Cells were recovered by centrifugation and suspended again in a fresh aliquot of the HBSS digestion solution for 15 minutes at 37 °C. The liberated cells were obtained through a 40μm mesh sieve and recovered and washed with RPMI-1640 medium. They were further separated on a Ficoll-Paque gradient to remove dead cells and the left cells were used for cytometric analysis. Anti-mouse-NKp46-Perep (eBioscience) was used to identify NK cells. Cells then were stained with Alexa Fluor647-conjugated anti-IFN-γ mAb according to the manufacturer’s instruction. Samples were analyzed by a BD FACS Aria flow cytometer.

### Antibody-mediated depletion of diverse cells

Anti-IFN-γ mAb (R4-6A2) was employed to deplete IFN-γ by intraperitoneal injection of 0.5 mg before subcutaneous administration and then 0.25 mg per 2 days. Rabbit anti-asialoGM1 antiserum (Wako Pure Chemical Industries, Ltd) was employed to deplete NK cells with dose of 20 μl in the same manner and schedule.

### NK activity assay

NK cytolytic activity was determined using mice splenocyte as effector and YAC-1 cells as target cells at different effector/target (E/T) ratios following manufacturer instruction. Target cells were labeled with ^51^Cr isotope. The percentage of specific lysis was calculated by the following formula: percent cytotoxicity = [(experimental release - spontaneous release by effector and target)/(maximal release - spontaneous release)] × 100. All assays were performed in triplicate.

### Real-time PCR

Total RNA was isolated by using Trizol reagent (Invitrogen) according to the manufacturer’s specifications. cDNA was reverse-transcribed using the Revert Aid RT-PCR system (Fermentas, Pittsburgh, PA, USA). Real-time PCR was performed by mixing cDNA with primers and Maxima SYBR Green qPCR Master Mix (Applied Biosystems, Carlsbad, CA, USA) using a Stratagene Mx3000P Real-time PCR System with supplied software (Applied Biosystems), according to the manufacturer’s instructions. The sequences of the primers were as follows: Sirt1(forward: 5′-GCTGACGACTTCGACGACG-3′; reverse: 5′-TCGGTCAACAGGAGGTTGTCT − 3′), PCNA (forward: 5′-TTTGAGGCACGCCTGATCC-3′; reverse: 5′-GGAGACG TGAGACGAGTCCAT-3′), Caspase3 (forward: 5′-ATGGAGAACAACAAAACCTC AGT-3′; reverse: 5′-TTGCTCCCATGTATGGTCTTTAC-3′), CXCL10 (forward: 5′-CCAAGTGCTGCCGTCATTTTC-3′; reverse: 5′-GGCTCGCA GGGATGATTTC AA-3′) and β-action (forward: 5′-GGCTGTATTCCCCTCCATCG-3′ reverse: 5′-CCAGTTGGTAACAATGCCATGT-3′). β-action was used as internal control for RNA integrity and loading normalization.

### Immunohistochemistry evaluation and TUNEL staining

Tumor tissue sections were suffered deparaffinage in xylene, and then rehydrated in a graded alcohol series and blocked with 3% H_2_O_2_ or 5% BSA in methanol for 10–30 minutes. Tissue sections were washed with PBS and then immunostained with primary antibodies for Ki-67 (Abcam) or CD49b (Biolegend). TUNEL staining (Calbiochem) was used to assess the apoptosis level according to the manufacturer’s instructions. After glass slides were mounted, each sample was observed at a 200 × magnification of microscopic field in 10 randomly selected areas.

### Western blotting analysis

Cells or tissues were lysed in RIPA lysis buffer (Beyotime) with 1 mM PMSF. The whole-cell lysates were subjected to SDS–PAGE. The membranes were incubated with specific primary antibodies against PCNA, Caspase-3, Cleaved Caspase-3 or β-actin antibody (both from Abcam, Cambridge, UK), followed by incubation with horseradish peroxidase-conjugated secondary antibodies (Hangzhou HuaAn Biotech, Zhejiang, China). Signals were visualized by chemiluminescent detection (Beyotime).

### Enzyme linked immunosorbent assay (ELISA)

ELISA assays were performed with a commercial ELISA kit (R&D Systems). The MSCs conditioned medium was collected for further experiment. Assays were performed in duplicate, and readings were compared with standard curves obtained with standard protein provided with the kit. Means and standard deviations of concentrations in triplicate samples were compared by t-test.

### Transwell assay

The chemotactic effect of MSCs overexpressed Sirt1 on NK cells were assayed using transwell with polycarbonate membranes (5 μm pore size, Cell Biolabs, San Diego, CA) in 24-well plate. NK cells (1.5 × 10^5^) were added in the upper chamber containing 200 μL serum-free medium, and 300μL MSCs-Sirt1 conditioned medium without serum was placed in the lower chamber. The plate was incubated at 37 °C in a humidified atmosphere containing 5% CO_2_ for 4 hours. At the end of the experiment, the cells that passed through the filters were counted under a microscope. These experiments were performed in triplicate.

## Statistical analysis

All of the experiments were repeated at least three times. Final data were expressed as mean ± standard deviation (SD). Statistical analysis of the data was done by using GraphPad Prism 5. Student’s t-test was used to compare between mean values of two groups. Value of at least p < 0.05 was considered statistically significant.

## Additional Information

**How to cite this article**: Yu, Y. *et al*. Mesenchymal stem cells with Sirt1 overexpression suppress breast tumor growth via chemokine-dependent natural killer cells recruitment. *Sci. Rep.* **6**, 35998; doi: 10.1038/srep35998 (2016).

**Publisher’s note:** Springer Nature remains neutral with regard to jurisdictional claims in published maps and institutional affiliations.

## Supplementary Material

Supplementary Information
