# Supplementary material for: Mesenchymal stem cells with Sirt1 overexpression suppress breast tumor growth via chemokine-dependent natural killer cells recruitment
Source: Sci Rep. 2016 Oct 26;6:35998. doi: 10.1038/srep35998 (PMC5080609; doi:10.1038/srep35998)
Supplement: Supplementary Information [file srep35998-s1.docx]

**Mesenchymal stem cells with Sirt1 overexpression suppress breast tumor growth via chemokine-dependent natural killer cells recruitment**

Yang Yu^1,a^, Yan Liu^2,a^, Chen Zong^3,a^, Qingbo Yu^4^, Xue Yang^3^, Lei Liang^3^, Fei Ye^3^, Li Nong^2^, Yuxian Jia^2^, Yongkui Lu^2,*^ & Zhipeng Han^3,*^

^1^Department of Urology, Affiliated Tumor Hospital of Guangxi Medical University, Nanning, People’s Republic of China;

^2^The Fifth Department of Chemotherapy, Affiliated Tumor Hospital of Guangxi Medical University, Nanning, People’s Republic of China;

^3^Tumor Immunology and Gene Therapy Center, Eastern Hepatobiliary Surgery Hospital, the Second Military Medical University, Shanghai, People’s Republic of China;

^4^Department of Thyroid Breast Surgery, Affiliated Hospital of Weifang Medical University, Weifang, People’s Republic of China;

^a^These authors contributed equally to the work;

*Corresponding author: Yongkui Lu, Tel: +86-771-5330700; Fax: +86-771-5308334; E-mail: [luyongkui616@126.com](mailto:luyongkui616@126.com) or Zhipeng Han, Tel: +86-21-81875331; Fax: +86-21-35030398; E-mail: [hanzhipeng0311@126.com](mailto:hanzhipeng0311@126.com).

**
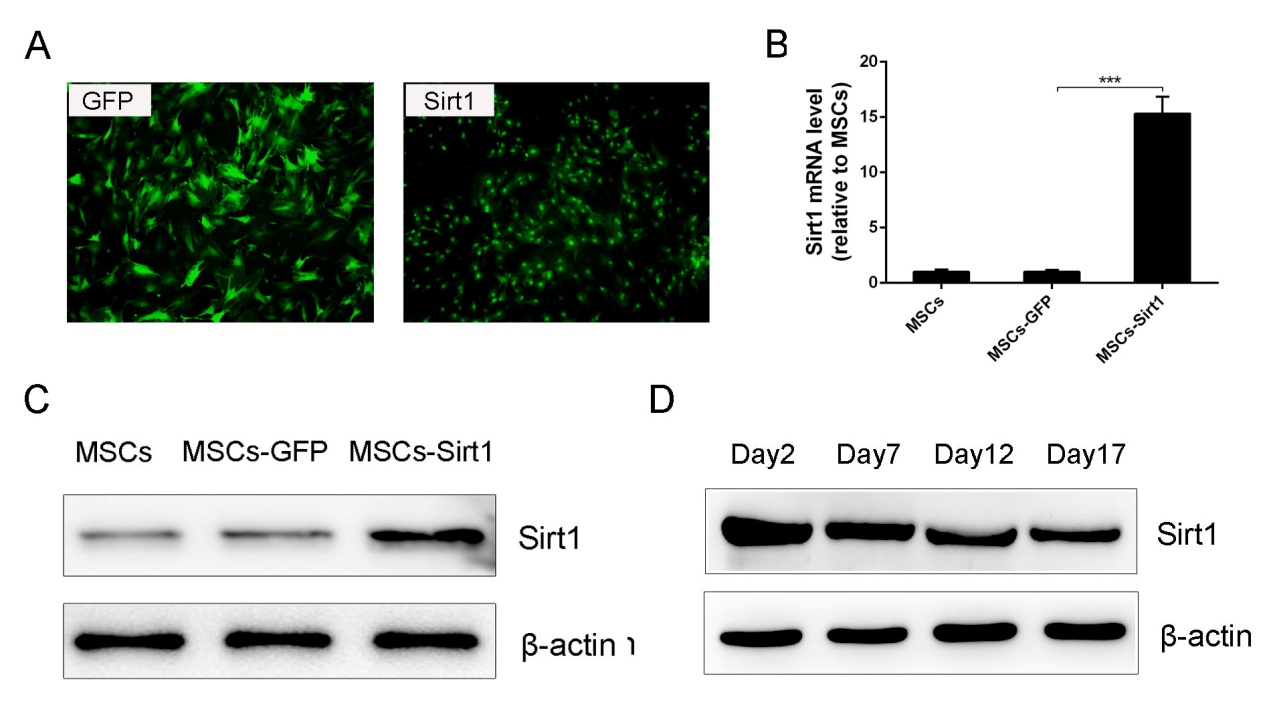
**

**Supplementary Fig.1 Sirt1 expression level of MSCs infected with an adenovirus containing the Sirt1 gene.** (A) MSCs were infected with an adenovirus containing GFP-tagged the Sirt1 gene and were detected by fluorescence microscope after infection 48h (original magnification: ×100). (B) Real-time PCR was employed to examine the expression level of Sirt1 in MSCs after infection 48h. The data were reported as ratio to MSCs. The data presented are from three replicates as mean ± SD. (C) Difference between Sirt1 expression of MSCs under Ad-GFP or Ad-Sirt1 treatment after infection 48h. (D) Changes about Sirt1 expression of MSCs-Sirt1 with time going. ***, P < 0.001.
